# How to Grow a Computational Biology Lab

**DOI:** 10.1371/journal.pcbi.1004397

**Published:** 2015-09-24

**Authors:** Alice Carolyn McHardy

**Affiliations:** Helmholtz Centre for Infection Research and Technical University of Braunschweig, Braunschweig, Germany; EMBL Heidelberg, GERMANY

## How the Lab Started

**Start of the lab:** 2007

**Size of the lab:** 16

**Research field:** Computational biology

I began building my lab after having been selected to run an independent junior research group by the Max Planck Society at the Max Planck Institute for Informatics in Saarbrücken in 2007. At a meeting at Rutgers University that summer, I convinced a very good postdoc, Ben Adams, who was working in Japan by that time, that joining my new lab would be a great idea. We arrived in Saarbrücken at about the same time in the fall, and that’s how the lab started. In the following year, I recruited another PhD student through advertising and three by contacting colleagues.

From 2009 onwards, I decided to hire on average two people per year, so that, assuming a stay of four years in the lab on average, the group would consist of 8–10 scientists at any given time, with different levels of seniority. Currently, the lab includes eight PhD students, several student programmers, interns, and nonscientific personnel, as well as two postdocs who just joined us. In 2010, we moved from Saarbrücken to Düsseldorf in Germany, as I was appointed to chair of Algorithmic Bioinformatics at Heinrich Heine University. In 2014, I accepted the position as the head of the Computational Biology of Infectious Diseases Lab at the Helmholtz Centre for Infection Research (HZI) in Braunschweig, Germany.

“*I decided to hire on average two people per year*.”

## Scientific Mission

One could define the core mission of the lab as developing methods and answering questions in biological sequence data analysis. We are focusing on problems in metagenomics and microbial community research, viral pathogen analysis, and phylogenetics, but we are also extending our interest to related topics involving sequence data analysis, such as the characterization of RNA-binding-protein binding sites from Photoactivatable-Ribonucleoside-Enhanced Crosslinking and Immunoprecipitation (PARCLIP) data and analyses of single-cell data. I think it is fascinating what kind of questions can be addressed by analysing biological sequence data. As we are now based at HZI, because of the centre’s research mission, our topics should be linked to research on human pathogens and their interactions with the host, which I personally also find a highly relevant mission. I also like to think that the scientific mission of the lab is to innovate and have fun in tackling challenging scientific questions in computational biology. If someone had a very new problem and could convince me that that he/she was capable of successfully investigating it and that the lab was the right place for this, I would be open to it being pursued.

“*The mission of the lab is to innovate and have fun*.”

## Time Management

If you become the head of a group and begin to teach courses, your workload increases gradually, and, at some point, you realize that you cannot handle this anymore, unless you work for 80 hours per week or more on a regular basis. Investigating the reasons why I had this workload by taking note of my activities over the day, not surprisingly, I found out that email had become a major time consumer. Since then, I have tried to reduce the amount of email writing, though with not as much success as I would have liked to have. Sometimes the issues of organization require a lot of communication with many different people, via email or otherwise. However, I try to avoid lengthy email conversations relating to scientific discussions and replace them with personal meetings, which are much more effective. Meetings with individual lab members are scheduled regularly, usually every 2–6 weeks, depending on personal preferences and project complexity, and more frequently when jointly preparing paper submissions, for instance. If I want to work on an article, grant, or a scientific problem, I make myself unavailable for half a day or a full day, as constant interruptions were another problem that prevented me from being productive. Teaching, grant writing, and paper writing take a lot of time, with teaching becoming less time consuming when you give the course for the second or third time and once you have the course material fully prepared. Be aware that if you begin to teach a course, preparing one hour of new lecture material might well take you 10–20 hours.

“*I make myself unavailable for half a day or a full day*.”

Saying yes to too many requests, be they scientific or otherwise, such as invitations to visit a colleague at another institution, acting as an external advisor for tenure requests, review invitations, and participating in grant writing or in grant evaluation panels, was another big time consumer, and I had to learn to say no. This is essential in order to continue to be productive. One has to choose wisely, as offending people in important positions by declining their requests is also not ideal and might mean trouble down the road. To give a specific example, I had since my arrival at my former institution participated in multiple grant-writing efforts but had taken a pick of the most sensible ones linking more closely to my research expertise. I had decided not to participate, for instance, in a grant proposal on research of Drosophila neuronal activities. By saying no to one of these proposal preparation efforts, I inadvertently offended a person in a higher position at my old institution, who then went on to complain to many other colleagues and at high management levels of our university (I heard this from three different sides) that I was unwilling to collaborate and had given him an “ice-cold” rejection. Efforts to mend this relationship from my side with involvement of others did not lead anywhere, and I was not being included by the same person subsequently in a proposal effort, which I would have suited well to and expressed interest in. As my colleagues were mostly supportive of me, there was no larger damage done, overall, as far as I could tell. Probably one cannot entirely avoid these kind of issues, unless one would like to spend all energy on fulfilling the expectations of others when joining a novel institution, rather than on efforts that seem sensible. One of the proposal efforts that I participated in was subsequently successful, and our group of principal investigators (PIs) was awarded an “excellence cluster”, which is one of the largest and most prestigious institutional research grants in Germany.

## Delegation of Work

Delegation of course work is also very important for coping with your responsibilities. Over the years, I have increasingly delegated more work, by jointly handling paper submissions, all kinds of administrative requests, invitations to review, filling out progress reports, and related things, either with my assistant or the group member working on the particular problem. I have been very lucky to have an excellent assistant, and this has made my life much easier. When we have been organizing a meeting, we have heard comments that this was the best-organized meeting participants had ever attended, although I only had a minor role in this. Another area in which I partly delegate is the supervision of bachelor’s and master’s theses, which is jointly done by myself and other lab members. I offer every student the possibility of regularly meeting with me to discuss their work; some make more use of this than others. Recently, I have begun to write grant applications with the students that are anticipated to work on the project, if they are already in the lab, following the suggestion of a colleague. I think this is sensible, as the student identifies more with the project and may also inject some creativity and it saves me some time.

## Motivation

From my experience people are usually highly motivated when they join the lab to work on a specific problem. If they are not, even though this may sound harsh, I think they probably should not have been hired in the first place. Motivation may drop if there are unanticipated difficulties in a project, in which case I am there to discuss and offer advice. Another problem can be conflicts between team members working together, which, luckily, has not happened very often. In this case, I have tried to mediate. Though this has not happened so far, in the most extreme case, if no satisfactory solution would be attainable, I would assign a person to a different project and try to find a replacement for the current project. Giving appropriate credit and positive feedback, encouraging creative and independent thinking, encouraging and supporting suggestions of how to improve the lab, delegating responsibilities to motivated lab members, and providing explicit recognition and visibility of the lab members’ contributions to the outside (e.g., in presentations) is likely to be motivating. When there is an accepted paper, the authors, including me, bring cake and drinks, and everyone enjoys this and congratulates the authors, so there is a within-group celebration and recognition for this achievement. Beyond that, I think a substantial part of motivation and excitement is an intrinsic personal quality and is also indicative of a match of the project to a person. Thus, in addition to the skill set, the motivation to work on a particular problem is a very important quality when I hire someone. Usually, this already becomes evident during the job interviews by the questions that are being asked by the applicant. If these are detailed and relate to the projects that I outline, I take this as a good sign, even more so if an applicant has already read some of the lab’s articles in preparation for the visit or for the initial application.

## How I Learned to Lead a Lab

In the beginning, I took part in several management courses, to learn how to best run a lab. Once I started to teach, I participated in a course training me how to do this effectively. In these courses, I received lots of advice and recommendations. In part, this has been very helpful from the beginning; however, hearing about how to run a research group was a bit abstract, and I could not immediately make use of these ideas in practice. On several occasions, I realized only after I had decided to follow a certain approach that this was very similar to a recommendation given to me. If I am uncertain on what might be the best approach to a particular question, I usually still get the advice and opinions of my colleagues at my university or experienced collaborators on the matter. Thus, my personal way of leading a research group has partly been based on advice and has partly been learning by doing.

## Communication inside the Lab

Within my lab, people are very open to each other and like to collaborate and communicate. I think that this communication between lab members is very important for exchanging knowledge and experiences; without it, the group would probably be less effective. A lot of the communication seems to be done during lunch time, when usually a few large groups go to lunch together. However, as there are at the moment group members still primarily based in Düsseldorf, others based in Braunschweig, and one group member working mostly in Munich, communication also often involves phone calls and Skype meetings. Everyone also visits the main lab location in Braunschweig on a regular basis, usually at least once a month, to allow for more in-depth personal interactions with the rest of the team and myself. I think such work settings and interactions will become more and more routine in the future, and so far it seems to work out well for us.

“*Group members are based in Düsseldorf, Braunschweig, and in Munich*.”

To further stimulate interactions, we have a weekly seminar, in which people present their projects or related scientific topics in a 45-minute presentation; sometimes, we also have invited guest speakers to present here. This seminar is made available via video conferencing to lab members at all locations, and they can also log in from home or abroad, if they are traveling. In the discussion section, which usually lasts 10–20 minutes, everyone is encouraged to give constructive feedback and also voice quite critical questions in these seminars. This means that the speaker may subsequently identify potential new avenues in a project, consider comments on presentation style and slides, or discover potential weak points in a presented analysis. We have recently started to rate talks, based on relevant criteria (novelty of presented material, clarity, and presentation style), and the winner of a semester series is announced subsequently. In addition to that, every six months, we have a literature seminar on a topic of special interest that lasts the entire day. The topic is decided on in advance in our group seminar, based on suggestions made either by myself or other group members. In our most successful seminars, we have chosen a number of articles from an article series or have selected multiple chapters from a book. Recently, we had our first two-day group retreat, at which people discussed several topics in smaller groups for a couple of hours, including also hands-on tutorials and little hacking sessions, in which, again, the relevant topics were jointly identified in advance. This event was quite popular and thus will likely be repeated. We also go for group dinners or activities every couple of months. The activities usually are hiking or bowling, as few people in the lab seem to share my enthusiasm for karaoke, and we have a democratic decision process installed for choosing the activity.

“*We have a literature seminar on a topic of special interest that lasts the entire day*.”

During working hours, it is also important that people have the possibility to work without being interrupted. As most people share their offices, people are therefore recommended to go to our meeting room for impromptu discussions over the day so as to not disturb other colleagues. Furthermore, lab members who want to escape from all interactions for a short period of time to concentrate fully on a project can work from home for part of the day.

## Advice to a Beginning Researcher

If I had to give advice to a potential PhD student, I might say that in considering whether to pursue a PhD, important factors to consider would be the degree of excitement about the potential research problem. The benefits that scientific work offers are the large degree of creative freedom, which one rarely encounters in other professions, and the satisfaction of contributing to advances on very important fundamental problems that are important to society and our planet. I might also say that one of the essential tasks for early career researchers is to produce research described in peer-reviewed articles over the course of the PhD and beyond. This is the most universally accepted and respected acknowledgment of these scientific results and makes the results of someone’s work officially become a contribution to science. To be able to follow through with this, given that one has decided on an interesting problem to pursue, it is helpful to think through the entire research plan, complete with all the important steps until the writing of the paper, and to not get sidetracked. Another point I assume that most early career researchers are aware of—but which cannot be stressed enough—is how important stays as a postdoctoral researchers abroad in one or more renowned laboratories are for a scientific career. In my own experience, I found that my time as a postdoctoral researcher at the IBM T. J. Watson Research Center in New York substantially broadened my horizon and expanded both my scientific and personal skill sets. Working in this renowned institution with mostly senior researchers from all over the world while living in the multicultural and bustling city of New York was a very interesting, new, and challenging experience that I will remember for my entire life and that I would not have wanted to miss.

“*Working at the IBM T. J. Watson Research Center and living in the multicultural city of New York was an experience*.”

